# The Liver Not Extirpated

**Published:** 1848-01

**Authors:** 


### THE LIVER NOT EXTIRPATED.

In our last number we gave an accout of the alleged extirpation of the liver by Dr. Thompson, of Columbia, Ohio, and as his patient was alive at the date of our latest accounts, eight days after the operation, we, of course, exprcssed the opinion, that the organ had not been removed, but that the mass was ovarian, and the liver, "*in a state of atrophy, from the pressure of the tumor, was pushed far up in the concave of the diaphragm, to which it might morbidly adhere.*" Before our article had passed through the press, but when it was too late to insert a postscript, a letter from Dr. Thompson, to the editor of the *Ohio Statesman*, met our eye, in which he announces the death of his patient, eleven days after the operation, and a post-mortem examination on the twelfth, which disclosed that "*the morbid mass was an ovarian tumor, which had occupied the greatly increased concavity of the diaphragm, carrying the liver before it;*" the organ being "*below the medium size, and a large portion of its convex surface adherent to the diaphragm.*"

We congratulate the Doctor, and all the gentlemen who advised or assisted in the operation, as well as the profession at large, on this happy disclosure. How the idea of extirpating the liver, could have entered the mind of a physician qualified and accustomed to perform surgical operations, is to us as mysterious as it is mortifying. Nevertheless, our science would have stood much worse had the operation actually been performed, than it does now. An operation, which from its nature must prove fatal, should, of course, never be performed.

